# Case report: Systemic lupus erythematosus combined with myocardial hypertrophy

**DOI:** 10.1002/iid3.1214

**Published:** 2024-03-27

**Authors:** Shanshan Wang, Xinfeng Wei, Wenqing Yang, Dan Zhang

**Affiliations:** ^1^ Innovative Institute of Chinese Medicine and Pharmacy Shandong University of Traditional Chinese Medicine Jinan China; ^2^ The Fifth People's Hospital of Jinan Jinan China; ^3^ Shandong Engineering Laboratory of Traditional Chinese Medicine Precise Therapy for Cardiovascular Diseases Jinan China; ^4^ Experimental Center Shandong University of Traditional Chinese Medicine Jinan China; ^5^ Key Laboratory of Traditional Chinese Medicine Classical Theory, Ministry of Education Shandong University of Traditional Chinese Medicine Jinan China

**Keywords:** myocardial hypertrophy, systemic lupus erythematosus

## Abstract

**Objective:**

Systemic lupus erythematosus (SLE) is a multisystem‐involved, highly heterogeneous autoimmune disease with diverse clinical manifestations. We report an extremely rare case of SLE with severe diffuse myocardial hypertrophy.

**Methods:**

The patientʼs echocardiography and cardiac magnetic resonance imaging (CMR) results indicated diffuse myocardial hypertrophy. After excluding coronary atherosclerosis, hypertensive cardiomyopathy, drug toxicity, and other causes, the patient was diagnosed with SLE‐specific cardiomyopathy. Medications such as hormones, antimalarials, immunosuppressants, and biologics were administered.

**Results:**

Ancillary test results were as follows: hs‐cTnI: 0.054 ng/mL (0–0.016); NTproBNP: 1594.0 pg/mL (<150); A contrast‐enhanced CMR revealed the diffuse thickening of the left ventricular wall with multiple abnormal enhancements, reduced left ventricular systolic and diastolic function, and moderate amount of pericardial effusion. Endomyocardial myocardial biopsy was performed, showing cardiomyocyte hypertrophy and degeneration, and no changes in myocarditis or amyloidosis. The pathology viewed by electron microscopy showed increased intracellular glycogen in the myocardium, and no hydroxychloroquine‐associated damage in the myocardium. The 24‐h ambulatory blood pressure and contrast‐enhanced computed tomography of coronary arteries were normal. The diagnosis of SLE‐specific cardiomyopathy was clear. The myocardial hypertrophy showed reversible alleviation following treatment with high‐dose corticosteroids. CMR results before and after treatment were as follows: interventricular septum, pretreatment (28) versus post‐treatment (22) mm; left ventricular inferior wall, pretreatment (18–21) versus post‐treatment (12–14) mm; left ventricular lateral wall, pretreatment (17–18) versus post‐treatment (10–12) mm; pericardial effusion (left ventricular lateral wall), pretreatment (25) versus post‐treatment (12) mm; left ventricular ejection fraction, pretreatment (38.9%) versus post‐treatment (66%).

**Conclusion:**

Myocardial hypertrophy may be an important sign of active and prognostic assessment in SLE diagnosis and management. Similarly, when encountering cases of myocardial hypertrophy, the possibility of autoimmune disease should be considered in addition to common causes.

## Highlights

- We report a rare case of severe diffuse myocardial hypertrophy directly caused by systemic lupus erythematosus (SLE), secondary to severe heart failure.
- Reversible improvement in myocardial hypertrophy occurred after high‐dose corticosteroid therapy.
- SLE is associated with a reversible form of cardiomyopathy.
- Myocardial hypertrophy may be an important sign of active and prognostic assessment of SLE.
- When encountering cases of myocardial hypertrophy, the possibility of autoimmune disease should be considered in addition to the common causes.

## 1 INTRODUCTION

Systemic lupus erythematosus (SLE) is a multisystem‐involved, highly heterogeneous autoimmune disease. Clinical manifestations of the multisystem involvement and immunological abnormalities (particularly positive antinuclear antibodies) are the characteristic features of SLE. The heart is most commonly affected in SLE, primarily characterized by pericarditis, myocarditis, valvulopathy, arrhythmia, and ischemic coronary artery disease; a few patients with SLE may develop cardiomyopathy. In this paper, we report a rare case of myocardial hypertrophy and subsequent severe heart failure directly caused by SLE.

## 2 CASE REPORT

A 43‐year‐old woman presented to the hospital in August 2023 with chest tightness, palpitations, breathlessness, and inability to lie down at night. In 2001, she was diagnosed with SLE. In 2006, she began to show chest tightness, palpitation, and decreased activity tolerance; echocardiography revealed myocardial hypertrophy and pericardial effusion. In 2021, cardiac magnetic resonance imaging (CMR) results showed symmetric hypertrophic cardiomyopathy with right ventricular myocardium involvement, multiple foci of delayed enhancement of the ventricular septum and left ventricular myocardium, and a small amount of pericardial effusion. Between 2001 and 2021, the SLE symptoms were active at times, while the cardiac disease was mild or severe. The patient was treated with prednisone (7.5–60 mg/day), hydroxychloroquine (0.2 g/day), mycophenolate mofetil (0.25–0.5 g/day), leflunomide (10 mg/day), cyclophosphamide (cumulative 10.6 g), bisoprolol (2.5 mg/day), and coenzyme Q10 (100 mg/day)  (Table 1 and 2).

**Table 1:** Comparison of echocardiography before and after treatment.

Echocardiography	March 24, 2023	August 29, 2023
Left atrium (mm)	39	35
Left ventricle (mm)	40	45
Interventricular septum (mm)	23	16
Left ventricular posterior wall (mm)	30	20
Right ventricle (mm)	24	23
Right atrial long diameter (mm)	41	38
Right atrial transverse diameter (mm)	35	36
Pericardial effusion (right ventricular anterior wall) (mm)	4.8	4
Pericardial effusion (left ventricular posterior wall) (mm)	25	8
left ventricular ejection fraction (LVEF) (%)	58	68
E/e'	15.4	9.1

**Table 2:** Comparison of CMR before and after treatment.

CMR	April 17, 2023	September 27, 2023
Left atrium (mm)	25	31
Left ventricle (mm)	48	48
Interventricular septum (mm)	28	22
Left ventricular inferior wall (mm)	18–21	12–14
Left ventricular lateral wall (mm)	17–18	10–12
Pericardial effusion (left ventricular lateral wall) (mm)	25	12
LVEF (%)	38.9	66

The patient was infected with the novel coronavirus in December 2022, which exacerbated her heart condition. Physical examination revealed a low respiratory sound in the left lower lung, regular heart rhythm, and a grade 2/6 systolic murmur audible in the heart apex under Valsava maneuver. Ancillary test results were as follows: hs‐cTnI: 0.054 ng/ml (0–0.016); NTproBNP: 1594.0 pg/mL (<150); d‐dimer: 0.96 ug/ml (FEU; <0.5); Antinuclear antibody profile: (xMAP) antinuclear antibody: 104.00 AU/mL (0–100); (indirect immunofluorescence) antinuclear antibody: granular 1:80 (<1:80); (xMAP) anti‐RNP antibody: 104.00 AU/ml (0–100); immunoglobulin E: 281.00 IU/ml (0–165); complement 3: 0.665 g/l (0.85–1.93); complement 4: 0.102 g/l (0.12–0.36). Blood and urine immunofixation electrophoresis and the antiphospholipid syndrome tests were negative. Echocardiography indicated cardiomyopathy, left ventricular hypertrophy, slight thickening of the right ventricular wall, enlargement of the left atrium, reduced left ventricular diastolic function, and pericardial effusion (moderate to large). An electrocardiogram showed sinus rhythm, low voltage in limb leads, abnormal Q waves, and ST‐T changes. The 24‐h ambulatory blood pressure was normal. Endomyocardial myocardial biopsy was performed, showing cardiomyocyte hypertrophy and degeneration, and no changes in myocarditis or amyloidosis; the immunofluorescence was negative for C3, C1q, IgA, IgM, and IgG. The pathology viewed by electron microscopy showed increased intracellular glycogen in the myocardium, no immune complex deposition in the microvessel wall, and no hydroxychloroquine‐associated damage in the myocardium. A contrast‐enhanced CMR revealed the diffuse thickening of the left ventricular wall with multiple abnormal enhancements, reduced left ventricular systolic and diastolic function, and moderate amount of pericardial effusion; nonischemic cardiomyopathy was considered in conjunction with the clinical findings. Contrast‐enhanced computed tomography of coronary arteries did not show any meaningful stenosis in the main segments of each coronary artery. Whole‐exome sequencing test results did not detect any variants related to the clinical phenotype of the subject. (Detailed disease progression, medications, and ancillary tests are described in Supporting Information: Materials 1–3.)

**Figure 1:**
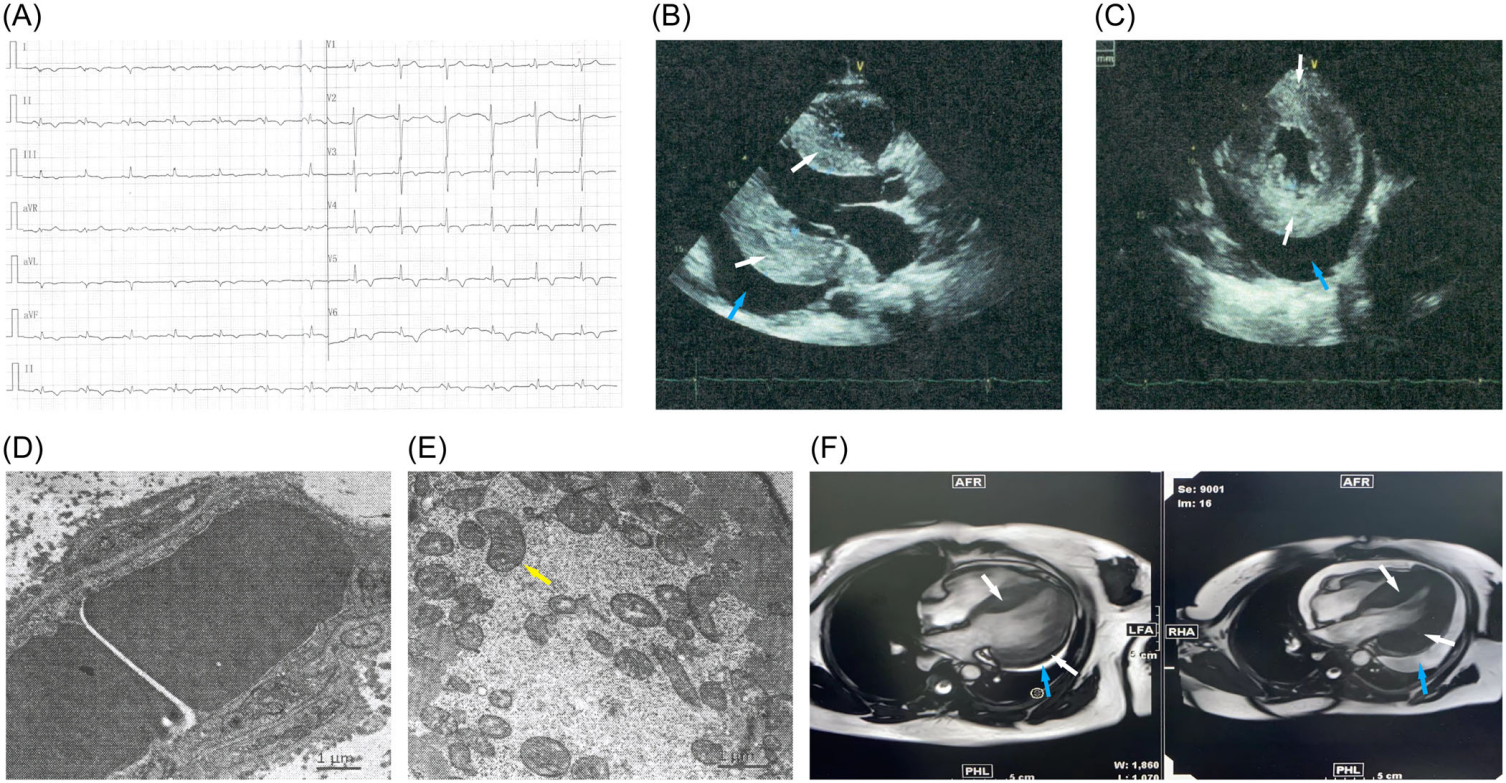
Auxiliary examination results: (A) electrocardiogram; (B) and (C) echocardiography showed myocardial hypertrophy (white arrows) and pericardial effusion (blue arrows); (D) and (E) endomyocardial myocardial biopsy (the pathology viewed by electron microscopy) showed increased intracellular glycogen in the myocardium with a yellow arrow pointing to mitochondria; (F) CMR results, 2023‐9‐27 for the former and 2023‐4‐18 for the latter, showed significant alleviation in myocardial hypertrophy (white arrows) and pericardial effusion (blue arrows).

Based on these examinations, the patient was diagnosed with lupus cardiomyopathy. In April 2023, drugs that inhibited myocardial remodeling and improved cardiac function were administered, including bisoprolol (2.5 mg/day), sacubitril valsartan sodium (12.5 mg/day), spironolactone (20 mg/day), furosemide (20 mg/day), and empagliflozin (5 mg/day); simultaneously, anti‐rheumatic treatment was strengthened with prednisone (30 mg/day) and cyclophosphamide (cumulative 20 g). In May 2023, belimumab (480 mg) was added. The symptoms of the patient did not alleviate significantly following medication. In August 2023, glucocorticoid pulse therapy was administered with methylprednisolone (1 g per day for 3 days) and mycophenolate mofetil (1.5 g/day) was added. In September 2023, the symptoms reduced significantly. Upon reexamination, NTproBNP: 869 pg/mL (0–125), immunoglobulins G, A, and M were normal, complement 3: 1.011 g/L (0.730–1.460), and complement 4: 0.187 g/L (0.100–0.400). Compared with a plain CMR scan and contrast enhancement in April 2023, the retested CMR contrast enhancement showed that the left ventricular wall was thinner, the left ventricular systolic and diastolic functions were improved, and pericardial effusion reduced, in comparison to those before treatment (Figure 1, Tables 1 and 2).

## 3 DISCUSSION

The most common cause of cardiomyopathy in patients with SLE may be the coexistence of hypertension and/or coronary artery disease^1^; hydroxychloroquine and corticosteroid therapy‐induced myocardial hypertrophy have also been reported. Myocardial hypertrophy directly caused by SLE has been reported less frequently. SLE is associated with a reversible form of cardiomyopathy.^2^ The SLE autoimmune processes are involved in cardiomyopathy pathogenesis.^3^, ^4^ Das and Cassidy^5^ detected anti‐cardiac antibodies in 20 of 32 patients with SLE and suggested that these antibodies could be involved in the development of lupus myocardial dysfunction. Jahns et al.^6^ suggested that based on individual genetic predisposition, heart‐directed autoimmune reactions could be because of cardiomyocyte damage induced by inflammation, ischemia, or exposure to cardiotoxic substances; apoptosis or necrosis of cardiomyocytes and the subsequent liberation of a “critical amount” of cardiac autoantigens may induce a self‐directed immune response and, in severe cases, perpetuate autoantibody‐mediated cardiac damage. Potentially pathogenic cardiac antibodies have been identified in dilated cardiomyopathy, such as cardio‐depressant,^7^ troponin I,^8^ myosin,^9^ muscarinic receptor type 2,^10^ and β1 receptor autoantibodies.^11^ Fiorito et al.^12^ suggested that hypertrophic obstructive cardiomyopathy was associated with genes in the human leukocyte antigen (HLA)‐DR region, and immunogenetic factors could be involved in the pathogenesis of the disease. Currently, the clinical diagnosis of myocardial involvement in SLE has no consensus.^13^ Electrocardiography, cardiac biomarkers, echocardiography, CMR, and endomyocardial muscle biopsy are the main diagnostic tools used clinically to assess myocardial involvement in SLE. Another useful diagnostic method is echocardiography or nuclear medicine to assess reversibility after a period of high‐dose corticosteroid therapy.^1^ Lupus‐specific cardiomyopathy should be considered when etiologies such as coronary atherosclerosis, hypertensive cardiomyopathy, and drug toxicity can be excluded.

After infection with the novel coronavirus, the patientʼs heart condition worsened, with varying degrees of myocardial damage and pericardial effusion. Through comprehensive multidisciplinary cooperation, after ruling out coronary artery disease, hypertension, side effects of drug therapy, myocardial amyloidosis, and antiphospholipid syndrome, combined with the reversibility of myocardial lesions following high‐dose glucocorticoid therapy, the diagnosis of lupus myocardial hypertrophy was made without considering the simultaneous coexistence of SLE and hypertrophic cardiomyopathy. This case is relatively rare in clinical practice.

## 4 CONCLUSION

SLE combined with myocardial involvement may be an important sign for activity and prognostic assessment in clinical diagnosis. We suggest that myocardial involvement should be included in the SLE disease activity index. When encountering cases of myocardial hypertrophy, autoimmune disease causes should be considered in addition to common causes.

## AUTHOR CONTRIBUTIONS

**Shanshan Wang**: Writing—original draft; investigation; resources. **Xinfeng Wei**: Investigation; resources. **Wenqing Yang**: Writing—review and editing; supervision. **Dan Zhang**: Writing—review and editing; supervision.

## CONFLICT OF INTEREST STATEMENT

The authors declare no conflict of interest.

## Supporting information

Supporting information.

Supporting information.

Supporting information.

Supporting information.

## Data Availability

The data that support the findings of this study are available from the corresponding author upon reasonable request.
