# Supplementary material for: Case report: Systemic lupus erythematosus combined with myocardial hypertrophy
Source: Immun Inflamm Dis. 2024 Mar 27;12(3):e1214. doi: 10.1002/iid3.1214 (PMC10966916; doi:10.1002/iid3.1214)
Supplement: Supplementary file 1 — Supporting information. [file IID3-12-e1214-s002.docx]

**Disease progression and medication**

| Date | Disease progression | Medication | |
| --- | --- | --- | --- |
|  |  | Systemic lupus erythematosus | Myocardial hypertrophy |
| 2001-2005 | ①Diagnosed with SLE | ①Prednisone 10mg qd po  ②Hydroxychloroquine 0.1g bid po (started in 2003) | None |
| 2006-2008 | ①Myocardial hypertrophy  ②Paroxysmal atrial fibrillation  ③Pericardial effusion (small amount) | ①Methylprednisolone 40mg qd ivdrip for 8 days → prednisone 30mg qd po→ prednisone 7.5mg qd po  ②Cyclophosphamide (cumulative 8g)  ③Hydroxychloroquine 0.1g bid po (discontinued in 2008 due to ocular lesions) | Amiodarone was used for 1 month and discontinued when the heart rhythm returned to the sinus. |
| 2009-2020 | ①Myocardial hypertrophy  ②Pericardial effusion (small to medium) | ①Prednisone 60mg qd po→15mg qd po  ②Mycophenolate mofetil 0.25g qd po  ③Leflunomide 10mg qd po  ④Cyclophosphamide (cumulative 10.6g) | None |
| 2021.1-2022.11 | ①Myocardial hypertrophy  ②Pericardial effusion (small amount) | ①Prednisone 15mg qd po  ②Mycophenolate mofetil 0.25g qd po  ③Leflunomide 10mg qd po | ①Bisoprolol 2.5mg qd po  ②Coenzyme Q10 100mg qd po |
| 2022.12-2023.2 | Infected with the novel coronavirus |  |  |
| 2023.3 | ①Myocardial hypertrophy  ②Pericardial effusion (medium to large) | ①Prednisone 15mg qd po  ②Cyclophosphamide (cumulative 11.6g) |  |
| 2023.4 | ①Myocardial hypertrophy  ②Pericardial effusion (medium amount) | ①Prednisone 30mg qd po  ②Cyclophosphamide (cumulative 20g)  ③Rivaroxaban 15mg qd po | ①Bisoprolol 2.5mg qd po  ②Sacubitril valsartan 12.5mg qd po  ③Spironolactone 20mg qd po  ④ Furosemide 20mg qd po  ⑤ Empagliflozin 5mg qd po |
| 2023.5 | ①Myocardial hypertrophy  ②Pericardial effusion (medium to large) | ①Prednisone 30mg qd po→25mg qd po  ②Belimumab 480mg ivdrip, 2 times |  |
| 2023.6-2023.7 | ①Myocardial hypertrophy  ②Pericardial effusion ( medium to large) | ①Prednisone 25mg qd po→20mg qd po  ②Blimumab 480mg ivdrip, 2 times |  |
| 2023.8-2023.9 | ①Myocardial hypertrophy  ②Pericardial effusion (small amount) | ①Methylprednisolone 1g qd ivdrip for 3 days  ②Prednisone 20mg qd po→15mg qd po  ③Elimumab 480mg ivdrip, 2 times  ④Mycophenolate mofetil 0.75g bid po |  |
| 2023.10 | ①Myocardial hypertrophy  ②Pericardial effusion (small amount) | ①Prednisone 15mg qd po→12.5mg qd po  ②Elimumab 480mg ivdrip, 1 time  ③Mycophenolate mofetil 0.75g bid po |  |

qd: once a day; bid: twice a day; ivdrip: intravenous drip; po: take medicine by mouth.
