# Supplementary material for: Case report: Systemic lupus erythematosus combined with myocardial hypertrophy
Source: Immun Inflamm Dis. 2024 Mar 27;12(3):e1214. doi: 10.1002/iid3.1214 (PMC10966916; doi:10.1002/iid3.1214)
Supplement: Supplementary file 2 — Supporting information. [file IID3-12-e1214-s003.docx]

**Laboratory tests**

| Date | Test items | Test results |
| --- | --- | --- |
| 2023.4 | Three items of myocardial infarction | hs-cTnI:0.054ng/ml↑[0-0.016], CK-MB:3.22ng/ml[0.3-4.0]，myoglobin:20.13 ng/ml [0-140.1]. |
|  | NT-proBNP | 1594.0pg/mL↑ [<150]. |
|  | Inflammatory factor | IL-1β, IL-2, IL-4, IL-5, IL-6, IL-8, IL-10, IL-12p70, IL-17, TNF-α, IFN-α, IFN-γ: normal. |
|  | Blood routine test | WBC: 2.92x10^9/L↓[3.5-9.5], absolute neutrophil count: 1.49x 10^9/L↓[1.80-6.3]，absolute lymphocyte count: 0.99x10^9/L↓ [1.1-3.2]，RBC：4.32x10^12/L[3.80-5.10]，Hb: 133.00g/L[115-150], PLT: 165x10^9/L[125-350]。 |
|  | Urine routine test | pH:8.0[4.5-8.0]，urine specific gravity:1.009[1.003-1.030]，Urine protein: negative. |
|  | Biochemical test | Albumin:35.9g/L↓ [40-55], K:3.69mmol/L[3.5-5.3], Na:142.83 mmol/L[137-147], glucose:3.62 mmol/L[3.58-6.05],creatinine: 44.30μmo1/L[41-81], GFR:118.83ml/min, Lipoprotein (a): 591.24mg/L↑[10-300], triglyceride:1.55 mmol/L[0.38-1.76], total cholesterol :4.66mmol/L[3.64-5.98], LDL-C: 2.92mmo1/L [<3.37], HDL-C:1.15mmo1/L[0.7-1.59]. |
|  | Iron metabolism | Serum iron:18.53µmol/L [9-27], total iron-binding capacity: 31.69µmol/L↓ [54-77], transferrin saturation:58.47%↑ [25-35], ferritin:496.11ng/ml↑ [10-120], transferrin:1.80g/L↓ [2-3.6], soluble transferrin receptor:14.97nmol/L [5.19-21.92]. |
|  | Coagulation | D-dimer:0.96ug/m1(FEU)↑ [<0.5]. |
|  | Blood and urine immunofixation electrophoresis | IgG κ-type M protein, IgG λ-type M protein, IgA κ-type M protein, IgA λ-type M protein, IgM κ-type M protein, IgM λ-type M protein, light-chain κ-type M protein, light-chain λ-type M protein, heavy-chain M protein: negative. |
|  | Immunoglobulin light chain | Immunoglobulin light chain κ: 2.700 g/L [1.7-3.7], immunoglobulin light chain λ: 1.880 g/L [0.9-2.1], light chain κ/light chain λ ratio:1.436 [1.35-2.65], free light chain κ:23.800 mg/L↑ [6.7-22.4], free light chain λ:23.800 mg/ L [8.3-27], free light chain κ-type/free light chain λ-type ratio:1.000 [0.31-1.56]. |
|  | Antinuclear antibody spectrum | (xMAP) anti-double-stranded DNA antibody, (xMAP) anti-SSA antibody, (xMAP) anti-SSB antibody, (xMAP) anti-Sm antibody, (xMAP) anti-Jo-1 antibody, (xMAP) anti-Ro-52 antibody, (xMAP) anti-Scl-70 antibody, (xMAP) anti-PM-Scl antibody, (xMAP) anti-ribosome antibody, (xMAP)anti-proliferating cell nuclear antigen antibody, (xMAP)anti-nucleosome antibody, (xMAP)anti-histone antibody, (xMAP) anti-attachment B antibody, (xMAP)anti-mitochondrial antibody M2 subtype: negative. (xMAP) anti-nuclear antibody: 104.00 AU/ml↑ [0-100], (IIF) anti-nuclear antibody: granular 1:80 [<1:80], (IIF) anti-double-stranded DNA antibody: 1:10 [<1:10], (xMAP) anti-RNP antibody: 104.00 AU/ml↑ [0-100]. |
|  | Immunoglobulin and complement | Immunoglobulins G, A, and M: normal, immunoglobulin E: 281.00 IU/mL↑ [0-165], complement 3: 0.665 g/L↓ [0.85-1.93], and complement 4: 0.102 g/L ↓ [0.12-0.36]. |
|  | Antiphospholipid syndrome test | Lupus anticoagulant (DRVVT), lupus anticoagulant (SCT), anticardiolipin antibody IgG, anticardiolipin antibody IgM, anti-beta2 glycoprotein 1 IgG, anti-beta2 glycoprotein 1 IgM: negative. |
|  | Three items of vasculitis | Anti-protease 3 antibody, anti-glomerular basement membrane antibody, anti-myeloperoxidase antibody: negative. |
|  | Thyroid function | Thyroid peroxidase antibodies, free triiodothyronine, free thyroxine, total triiodothyronine, total thyroxine, thyrotropin, thyroglobulin antibodies, thyrotropin receptor antibodies: normal. |
|  | Aldosterone, renin（supine position） | Aldosterone:26.7ng/dl↑ [3.0-23.6], renin:36.3uIU/ml [2.8-39.9], aldosterone/renin:0.736 [<3.7]. |
| 2023.7 | hs-cTnT | 44.82pg/ml↑ [0-15]. |
|  | Myocardial enzyme | CK-MB: 7.2ng/ml↑ [0.3-4.0], LDH:273U/L↑ [120-230]. |
| 2023.9 | NT-proBNP | 869pg/ml↑ [0-125]. |
|  | Immunoglobulin and complement | Immunoglobulin G, A, M: normal, complement 3: 1.011g/L [0.730-1.460], complement 4: 0.187g/L [0.100-0.400]. |
|  | Blood routine test | WBC, RBC, Hb, PLT: normal. |
|  | Erythrocyte sedimentation rate, hs-CRP | Normal. |
