# Supplementary material for: Case report: Systemic lupus erythematosus combined with myocardial hypertrophy
Source: Immun Inflamm Dis. 2024 Mar 27;12(3):e1214. doi: 10.1002/iid3.1214 (PMC10966916; doi:10.1002/iid3.1214)
Supplement: Supplementary file 3 — Supporting information. [file IID3-12-e1214-s004.docx]

**Imaging and other ancillary tests**

1. **Date:** March 2023

**Test items:** Echocardiography

**Test results:** Ascending aorta:27mm, left atrium:39mm, left ventricle:40mm, interventricular septum:23mm, posterior wall of left ventricle:30mm, lateral wall of left ventricle:23mm, anterior wall of left ventricle:16mm, inferior wall of left ventricle:20mm, right ventricle:24mm, longitudinal diameter of right atrial:41mm, transverse diameter of right atrium:35mm, main pulmonary artery:26m, and the thickness of the right ventricular free wall was 7mm. Fluid dark areas were detected in the pericardial cavity, with a thickness of about 4.8 mm at the anterior wall of the right ventricle, 25 mm at the posterior wall of the left ventricle, 10 mm at the lateral wall of the right ventricle, 12 mm at the top of the right atrium, 22 mm at the lateral wall of the left ventricle, and 4 mm at the apical part of the heart. There was a spotty and patchy echogenicity in the myocardial hypertrophy of the left ventricle. LVEF: 58%, E/e':15.4.

Ultrasonography Diagnosis: cardiomyopathy; left ventricular hypertrophy and slight thickening of the right ventricular wall; left atrial enlargement; mitral regurgitation (mild-moderate); aortic regurgitation (mild); tricuspid regurgitation (mild); pulmonic regurgitation (mild); left ventricular diastolic hypoplasia; and pericardial effusion (medium to large).

2. **Date:** March 2023

**Test items:** Ultrasound myocardial strain imaging

**Test results:** LVGLS: -4.5%, overall long-axis strain in the left ventricle decreased.

3. **Date:** April 2023

**Test items:** Electrocardiogram

**Test results:** Sinus rhythm, low voltage in limb leads, abnormal Q waves, ST-T changes.

**4. Date: April 2023**

**Test items:** Echocardiography

**Test results:** Left atrium: 37mm, left ventricle: 41mm, right ventricle: 25mm, interventricular septum: 20mm, posterior wall of the left ventricle: 26mm, LVEF: 58%, shortening of the left ventricle short-axis: 32%, E/e': 17, interventricular septum and the left ventricular wall were uniformly thickened, and the wall echoes were dense and rough. The systolic amplitude of the septum and left ventricular wall was still normal, with limited diastolic function. No significant narrowing of the left ventricular outflow tract was seen. Mitral valve closure was suboptimal. Fluid dark areas were detected in the pericardial cavity, approximately 27 mm in the posterior wall of the left ventricle, 7 mm in the anterior right ventricle, 16 mm in the lateral wall of the left ventricle, and 10 mm in the right paraventricular space. Doppler examination: the blood flow rate in the left ventricular outflow tract was normal, and there was no significant pressure difference. A small amount of regurgitation was seen in the mitral valve.

Ultrasound diagnosis: myocardial involvement, hypertrophic nonobstructive cardiomyopathy? Myocardial amyloidosis? decreased left ventricular diastolic function, and moderate pericardial effusion.

**5. Date:** April 2023

**Test items:** 24-hour ambulatory blood pressure

**Test results:** All-day average: 120/75mmHg, daytime average: 122/77mmHg, nighttime average: 114/70mmHg, early morning blood pressure: 119/76mmHg.

**6. Date:** April 2023

**Test items:** Endomyocardial Biopsy

**Test results:** Biopsy Pathology Report: (Right ventricular septum endomyocardial biopsy) Cardiomyocyte hypertrophy, degeneration, no myocarditis or amyloidosis changes were seen. Immunofluorescence results: C3 (-), C1q (-), IgA (-), IgM (-), IgG (-). Electron microscopy pathology: increased intracellular glycogen in the myocardium, no immune complex deposition morphology in the microvessel wall, and no hydroxychloroquine-associated damage in the myocardium.

**7. Date:** April 2023

**Test items:** Ambulatory electrocardiogram

**Test results:** Sinus rhythm; episodic atrial premature beats; episodic ventricular premature beats, some intermittent; T-wave alterations; heart rate variability: SD >50ms.

**8. Date:** April 2023

**Test items:** Ultrasound of adrenal glands and renal arteries

**Test results:** No significant masses were detected in the bilateral adrenal region, and color Doppler showed no abnormal blood flow signals. The resistance index of the right renal artery was increased.

**9. Date:** April 2023

**Test items:** CMR plain scan

**Test results:** The anteroposterior diameter of the left atrium was 25 mm, and the transverse diameter of the left ventricle was 48 mm. The left ventricular wall was diffusely thickened, with the thickest part about 28mm, located in the proximal segment of the inferior septum, 18-21mm in the inferior wall, and 17-18mm in the lateral wall. The overall contractile function of the left ventricle was reduced, the diastolic compliance of the thickened myocardium was reduced, the left ventricular wall was characterized by multiple patches of long T1 and long T2 signals, the left ventricular outflow tract was patent, and there was no systolic "SAM" sign in the mitral valve. The right atrium and right ventricle were not large, the right ventricular systolic motion was normal, and the right ventricular wall did not show significant thickening. There was moderate effusion in the pericardial cavity, and the left ventricular lateral wall effusion was 25 mm wide. The internal diameter of the main pulmonary artery was about 26 mm, and the internal diameter of the ascending aorta at the same level was about 28 mm. Left heart function: LVEF 38.9%, C0 3.4L/min, EDV 127.8ml, EDVi 84.5ml/m^2^.

Impressions: Diffuse thickening of the left ventricular wall with multiple abnormal signals, reduced left ventricular systolic and diastolic function; moderate pericardial effusion.

**10. Date: April 2023**

**Test items:** CMR contrast enhancement

**Test results:** Myocardial first-pass perfusion showed scattered small areas of patchy hypoperfusion between the left ventricular muscle walls; On delayed scanning, the left ventricular wall was widely enhanced with heterogeneous signals, and additional nonenhanced nodular shadows were seen in the lateral wall of the left ventricle and in the mid-septum of the inferior septum.

Impression: diffuse thickening of the left ventricular wall with multiple abnormal enhancements, left ventricular systolic and diastolic hypoplasia, and moderate pericardial effusion. Consider non-ischemic cardiomyopathy in conjunction with clinical findings, including non-obstructive hypertrophic cardiomyopathy and systemic lupus erythematosus cardiac damage (complex).

**11. Date:** April 2023

**Test items:** Contrast-enhanced CT of the whole aorta

**Test results:** Aorta and its main branches did not show stenosis or dilatation changes; few calcifications of the coronary arteries; biventricular wall thickening, uneven myocardial density in the interventricular septum and left ventricular free wall, and a large amount of pericardial effusion; uneven perfusion in both lungs and incomplete lung expansion in the left lung part.

**12. Date:** April 2023

**Test items:** Contrast-enhanced CT of coronary artery

**Test results:** Calcification of anterior descending coronary artery; the coronary arteries are of the left dominant type; there is no meaningful stenosis in the main segments of the coronary arteries. CT findings suggest myocardial involvement; calcification of the mitral annulus; moderate amount of pericardial effusion.

**13. Date:** May 2023

**Test items:** Whole Exome Sequencing

**Test results:**

Main results: No pathogenic/suspected pathogenic variants/unspecified variants of clinical significance with matching inheritance patterns were detected in relation to the clinical phenotype of the subjects.

Secondary results: (i) One variant of undetermined significance was detected in the LZTR1 gene associated with Noonan syndrome type 10/Noonan syndrome type 2/ Neurinomatosis type 2 susceptibility, which was partially associated with the subject's phenotype. (ii) One variant of undetermined significance was detected in the PON2 gene associated with coronary artery disease susceptibility, which was partially associated with the subject's phenotype.

Mitochondrial gene test results: No mitochondrial gene variants associated with clinical phenotype were detected.

Chromosome CNV/Other Supplementary Report Results: No pathogenic chromosome CNV variants above 1M and no LOH variants above 5M associated with the phenotype of the prior subject were detected by high-throughput data information analysis.

**14. Date:** May 2023

**Test items:** Echocardiography

**Test results:** Ascending aorta:27mm, left atrium:36mm, left ventricle:40mm, interventricular septum:22mm, posterior wall of left ventricle:28mm, lateral wall of left ventricle:22mm, anterior wall of left ventricle:17mm, inferior wall of left ventricle: 19mm, right ventricle:24mm, longitudinal diameter of right atrium:43mm, transverse diameter of right atrium:35mm, main pulmonary artery:26mm, and free wall of right ventricle:7.9mm. LVEF:58%, E/e':12.2. Fluid dark areas were detected in the pericardial cavity, with a thickness of about 8.5mm at the anterior wall of the right ventricle, 24.6mm at the posterior wall of the left ventricle, 11.0mm at the lateral wall of the right ventricle, 7.6mm at the top of the right atrium, 20.6mm at the lateral wall of the left ventricle, and 4.0mm at the apical part of the heart. The point of strong echogenicity was seen in the myocardial hypertrophy of the left ventricle.

Ultrasound diagnosis: cardiomyopathy; left ventricular hypertrophy, slightly thickened right ventricular wall; left atrial enlargement; mitral regurgitation (mild-moderate); aortic regurgitation (mild); tricuspid regurgitation (mild); pulmonic regurgitation (mild); left ventricular diastolic function decreased; and pericardial effusion (medium to large).

**15. Date:** May 2023

**Test items:** Ultrasound myocardial strain imaging

**Test results:** LVGLS: -5.1%, overall long-axis strain in the left ventricle significantly decreased.

**16. Date:** June 2023

**Test items:** Echocardiography

**Test results:** Ascending aorta:26mm, left atrium:35mm, left ventricle:41mm, interventricular septum:23mm, posterior wall of left ventricle:28mm, lateral wall of left ventricle:26mm, anterior wall of left ventricle:22mm, inferior wall of left ventricle:18mm, right ventricle:24mm, longitudinal diameter of right atrial:38mm, transverse diameter of right atrium:31mm, main pulmonary artery:28mm, right ventricular free wall:8.4mm. LVEF:55%, E/e':15.7. Fluid dark areas were detected in the pericardial cavity, with a thickness of about 7mm at the anterior wall of the right ventricle, 25mm at the posterior wall of the left ventricle, 9mm at the lateral wall of the right ventricle, 8mm at the right atrial collar, 16mm at the lateral wall of the left ventricle, and 4mm at the apical region. The left ventricular hypertrophic myocardium was characterized by strong echoes in spots and patches, especially in the interventricular septum and the left ventricular inferior wall.

Ultrasound diagnosis: myocardial lesions; left ventricular hypertrophy and slight thickening of the right ventricular wall; slight widening of the main pulmonary artery; mitral sclerosis with regurgitation (mild); aortic regurgitation (mild); tricuspid regurgitation (mild); increased left ventricular filling pressures; and pericardial effusion (moderate to large).

**17. Date:** June 2023

**Test items:** Ultrasound myocardial strain imaging

**Test results:** LVGLS: -6.2%, overall long-axis strain in the left ventricle significantly decreased.

**18. Date:** August 2023

**Test items:** Echocardiography

**Test results:** Ascending aorta:28mm, left atrium:35mm, left ventricle:45mm, interventricular septum:16mm, left ventricular posterior wall:20mm, right ventricle: 23mm, longitudinal diameter of the right atrium:38mm, transverse diameter of the right atrium:36mm, main pulmonary artery:28mm. LVEF:68%, E/e':9.1. Fluid dark areas were detected in the pericardial cavity, with a thickness of about 4mm at the anterior wall of the right ventricle, and about 8mm at the posterior wall of the left ventricle.

Ultrasound diagnosis: cardiomyopathy (infiltrative cardiomyopathy is highly likely); left ventricular hypertrophy; main pulmonary artery widening; mitral regurgitation (mild); aortic regurgitation (mild); tricuspid regurgitation (mild); left ventricular filling abnormality; pericardial effusion (small amount).

**19. Date:** September 2023

**Test items:** CMR contrast enhancement

**Test results:** In contrast to the CMR plain scan and contrast enhancement in April 2023, the internal diameters of the left atrium and left ventricle were not large (left atrium 31m, former 25m; left ventricle 48m, same as before); the left ventricular wall was diffusely thickened (the thickest part was located in the upper septum, about 22mm, former 28mm; the lower wall was 12-14mm, former 18-21mm; and the lateral wall was 10-12m, former 17-18mm); the overall systolic function of the left ventricle was reduced, and diastolic compliance of the thickened myocardium was reduced. The left ventricular wall was characterized by multiple patches of long T1 and long T2 signals; the left ventricular outflow tract was patent, and there was no "SAM" sign in the mitral valve. Opening and closing of the aortic valve was normal, and there was no significant regurgitant signal. The right atrium and right ventricle were not large, the right ventricular systolic motion was normal, and the right ventricular wall was not significantly thickened. Opening and closing of the tricuspid valve was normal, with no significant regurgitant signal. There was no pericardial thickening; the pericardial cavity had a moderate amount of effusion, and the left ventricular lateral wall effusion was 12 mm wide, former 25 mm. The internal diameter of the main pulmonary artery was about 30m, former 26mm, and the internal diameter of the ascending aorta at the same level was about 30m, former 28. Left heart function: LVEF 66% (former 38.9%), C0 6.1L/min, EDV 143.9ml, EDVi 89.3m1/m. Myocardial first-pass perfusion showed scattered small patchy areas of hypoperfusion between the left ventricular muscle walls, as before; delayed scan showed extensive enhancement of the left ventricular wall with heterogeneous signals, and additional non-enhancing nodular shadows in the left ventricular lateral wall and inferior septal mid-septum.

Impression: Compared with the CMR plain scan and contrast enhancement in April 2023: the left ventricular wall was thinner than before, left ventricular systolic and diastolic function was better than before, there were no significant changes in the multiple abnormal enhancements, and pericardial effusion was less than before.
