# Supplementary material for: Case report: Systemic lupus erythematosus combined with myocardial hypertrophy
Source: Immun Inflamm Dis. 2024 Mar 27;12(3):e1214. doi: 10.1002/iid3.1214 (PMC10966916; doi:10.1002/iid3.1214)
Supplement: Supplementary file 4 — Supporting information. [file IID3-12-e1214-s001.docx]

November 1, 2023

Dear Editor:

We wish to submit an original research article entitled “Case report: a case of systemic lupus erythematosus combined with myocardial hypertrophy” for consideration for publication by Joint Bone Spine.

In this paper, we report a scarce case of SLE with severe diffuse myocardial hypertrophy. The patient's echocardiography and cardiac magnetic resonance imaging indicated diffuse myocardial hypertrophy. After excluding coronary atherosclerosis, hypertensive cardiomyopathy, drug toxicity, and other causes, the patient was diagnosed with SLE-specific cardiomyopathy. Reversible improvement in myocardial hypertrophy occurred after high-dose corticosteroid therapy. Myocardial hypertrophy may be an important sign of active and prognostic assessment in the diagnosis and management of SLE. Similarly, when encountering cases of myocardial hypertrophy, the possibility of autoimmune disease should be considered in addition to the common causes. These findings are likely to be of great interest to clinicians reading your journal.

We confirm that this work is original and has not been previously published, and is not currently under consideration for publication elsewhere. All authors have contributed significantly and have read and approved the submitted work.

Thank you for your consideration of this manuscript. We look forward to your response.

Yours sincerely.

Dr. Wenqing Yang

wenqing-yang@hotmail.com
